# Small molecule inhibitors of the *Yersinia *type III secretion system impair the development of *Chlamydia *after entry into host cells

**DOI:** 10.1186/1471-2180-9-75

**Published:** 2009-04-21

**Authors:** Sandra Muschiol, Staffan Normark, Birgitta Henriques-Normark, Agathe Subtil

**Affiliations:** 1 Institut Pasteur, Unité de Biologie des Interactions Cellulaires, CNRS URA 2582, Paris, France; 2 Swedish Institute for Infectious Disease Control, SE-171 82 Solna, Sweden; 3 Department of Microbiology, Tumor and Cell Biology, Karolinska Institutet, SE-171 77 Stockholm, Sweden

## Abstract

**Background:**

*Chlamydiae *are obligate intracellular pathogens that possess a type III secretion system to deliver proteins into the host cell during infection. Small molecule inhibitors of type III secretion in *Yersinia*, termed INPs (Innate Pharmaceuticals AB) were reported to strongly inhibit *Chlamydia *growth in epithelial cells. In this study we have analyzed the effect of these drugs on bacterial invasiveness.

**Results:**

We demonstrate that INPs affect *Chlamydia *growth in a dose dependent manner after bacterial invasion. The efficiency of *C. trachomatis *L2 and *C. caviae *GPIC entry into host cells was not altered in the presence of INPs. In *C. caviae*, entry appears to proceed normally with recruitment of actin and the small GTPases Rac, Cdc42 and Arf6 to the site of bacterial entry.

**Conclusion:**

INPs have a strong inhibitory effect on *Chlamydia *growth. However, bacterial invasion is not altered in the presence of these drugs. In the light of these results, we discuss several hypotheses regarding the mode of action of INPs on type III secretion during the *Chlamydia *infectious cycle.

## Background

*Chlamydiae *are obligate intracellular pathogens with a complex developmental cycle. The first step is the attachment of the infectious form, the elementary body (EB), to a host cell. After entry, the bacteria differentiate into non-infectious reticulate bodies (RBs), which reside inside the host cell within a membrane-bound compartment, termed the inclusion. In this protected niche, RBs replicate and eventually differentiate into EBs, which, upon their release from the host cell, can start a new round of infection.

*Chlamydia*, like many other gram-negative pathogens, employ a type III secretion (T3S) system to deliver bacterial proteins into the host cell [1]. A large family of *Chlamydia*-specific proteins has been shown to be translocated by this process by RBs into the chlamydial inclusion membrane (Inc proteins) [2]. In addition, chlamydial effector proteins were also found to be secreted into the host cell cytoplasm during intracellular replication [3]. The function of most of the T3S substrates remains to be identified. Structural components of the type III secretion machinery have also been detected on EBs [4-6] and it has been shown that EBs possess functional secretion apparatuses [7].

Entry of *Chlamydia *into host cells requires the attachment of EBs to the host cell surface. A number of surface associated molecules and receptors have been described, suggesting that *Chlamydia *use multiple strategies for ensuring adhesion to the host cell [8]. Upon entry, *Chlamydia *induce actin rearrangements and small GTPases are recruited to the bacterial entry site [9-12]. Interestingly, the EB-associated T3S protein TARP (translocated actin recruiting phosphoprotein) has actin nucleating activity and is required for *Chlamydia *entry into host cells [13-16]. Other proteins might be translocated by T3S at the entry step, which remain to be identified. Importantly, EBs are metabolically inactive, and proteins that are translocated during the entry process have been synthesized during the previous infectious cycle and stored in the bacteria to be translocated upon contact with the host cell.

Recently, we and others have shown that small molecule inhibitors of the *Yersinia *type III secretion system, collectively termed INPs, disrupt the progression of the cycle of *Chlamydia *development [17-20]. In our previous study, we reported a partial effect of INPs on bacterial invasion, which was assessed by counting the number of inclusions present at 40 h post infection (p.i.) in cultures that were treated with drug for 3 h during infection. In order to clarify if this observed effect is due to the inhibition of bacterial invasion or to the inhibition of early events during the onset of *Chlamydia *development, we further examined the effect of INPs on *Chlamydia *entry.

## Results

### INPs affect *Chlamydia *development post entry

**Figure 1:**
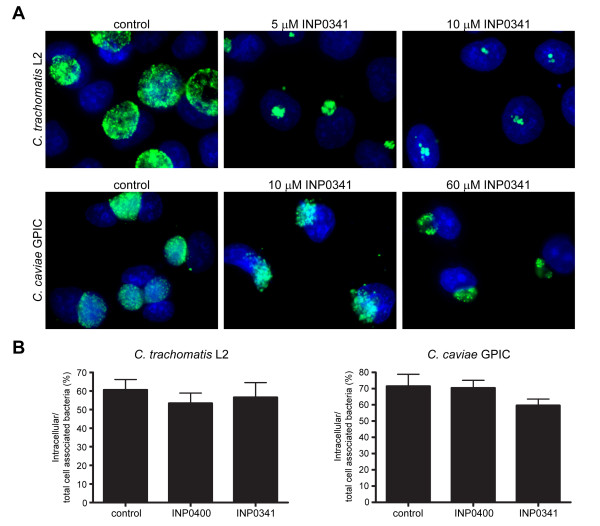
**Effect of INPs on *Chlamydia *intracellular development and entry**. (A) HeLa cells infected with *C. trachomatis *L2 (top) or *C. caviae *GPIC (bottom) were grown in the presence of INP0341 for 24 h at the concentrations indicated. After fixation, bacteria were labelled with anti-EfTu antibody (green) and host cell nuclei were stained with Hoechst 33342 (blue). (B) HeLa cells were infected with *C. trachomatis *L2 or *C. caviae *GPIC for 2.5 h in the presence or absence of 60 μM INP0400 or INP0341 and extracellular and intracellular bacteria were differentially immunolabelled as previously described [11]. The number of extra- and intracellular bacteria in untreated and treated cells were counted in 15 fields with an average of 75 bacteria per field. The efficiency of entry is expressed as the ratio of intracellular to total cell-associated bacteria (intracellular and extracellular). The data shown represent the average and the standard error of 30 fields from two independent experiments.

In our previous study, we used the small molecule INP0400, a derivative of salicylidene acylhydrazide identified as a specific inhibitor of *Y. pseudotuberculosis *T3S. We found that INP0400 progressively inhibited *C. trachomatis *L2 replication in doses from 5 to 25 μM [17]. In the present study we included another derivative of salicylidene acylhydrazide, INP0341. Dose response studies on chlamydial inclusion size showed that INP0341 was even more potent than INP0400 in inhibiting *C. trachomatis *L2 replication, as 10 μM INP0341 was already sufficient to strongly inhibit bacterial multiplication (Fig. 1A). We also tested the effect of these two INPs on the development of another strain of *Chlamydia*, *C. caviae *GPIC. At equivalent concentrations of INPs, the effect on inclusion size was always more pronounced on *C. trachomatis *than on *C. caviae *inclusions, suggesting that the latter strain is less susceptible to the drug (Fig. 1A). Treatment with 60 μM INP0341 resulted in a 99.8% reduction in the yield of infectious *C. caviae *EB particles. This reduction in infectivity is much greater than the decrease in inclusion size. It is consistent with the greater decrease in infectivity than inclusion size that we saw previously with INP0400 on *C. trachomatis *L2 [17]. In subsequent experiments we decided to use 60 μM of INPs, which fully inhibited development of *C. trachomatis *L2, and had a very strong effect on *C. caviae *multiplication.

In order to quantify the efficiency of *Chlamydia *entry in the presence of INPs, HeLa cells were infected with *C. trachomatis *L2 or *C. caviae *GPIC in the presence or absence of INP0400 or INP0341. At 2.5 h p.i. extracellular and intracellular bacteria in mock-treated (DMSO) or 60 μM INP-treated cultures were measured as previously described [11]. The efficiency of entry (intracellular/total cell associated bacteria) was quantified. INPs had no significant effect on *C. trachomatis *L2 and *C. caviae *GPIC invasion, when present during infection (Fig. 1B). Identical observations were made when the cells and/or the bacteria were preincubated for 15 minutes in the presence of INPs prior to infection or when bacteria were left to adhere on the cells at 4°C before allowing internalization to proceed at 37°C in the continuous presence of the drug (data not shown).

### Recruitment of actin and small GTPases to *Chlamydia *entry sites during infection in the presence of INPs

Although the overall efficiency of entry was not affected by INPs over a 2.5 h period of infection, a possibility remained that the bacteria used an alternative route of entry in the presence of the drug. To rule out this possibility, we observed some of the molecular events that accompany *Chlamydia *entry.

**Figure 2:**
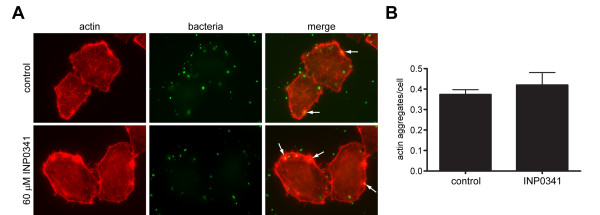
**Recruitment of actin to *C. caviae *GPIC entry sites**. HeLa cells were infected with FITC-labelled *C. caviae *GPIC in the presence or absence of 60 μM INP0341. At 10 minutes p.i. cells were fixed and actin filaments were visualized with Alexa-Fluor 546-phalloidin. (A) Actin remodelling around FITC-labelled bacteria was observed in control cells as well as in cells treated with INP0341 (arrows). (B) Quantification of actin aggregates in the presence or absence of INP0341. The number of actin aggregates per field was divided by the number of cells in the field (n>30). The average and standard deviation from three fields are shown.

Upon contact with host cells, *Chlamydia *activate small GTPases and induce actin polymerization [8]. These events are more pronounced in cells infected with *C. caviae *GPIC [11] than in cells infected with *C. trachomatis *L2 [10]; therefore we used the former. To synchronize infection, bacteria were centrifuged onto the cells and fixed 10 minutes after contact. *C. caviae *GPIC entry sites showed characteristic local actin rearrangements in control cells. Similar actin aggregates were observed in cells treated with INP0341 (Fig. 2A) or INP0400 (data not shown). The number of actin aggregates per cell was identical in treated and untreated samples (Fig. 2B).

**Figure 3:**
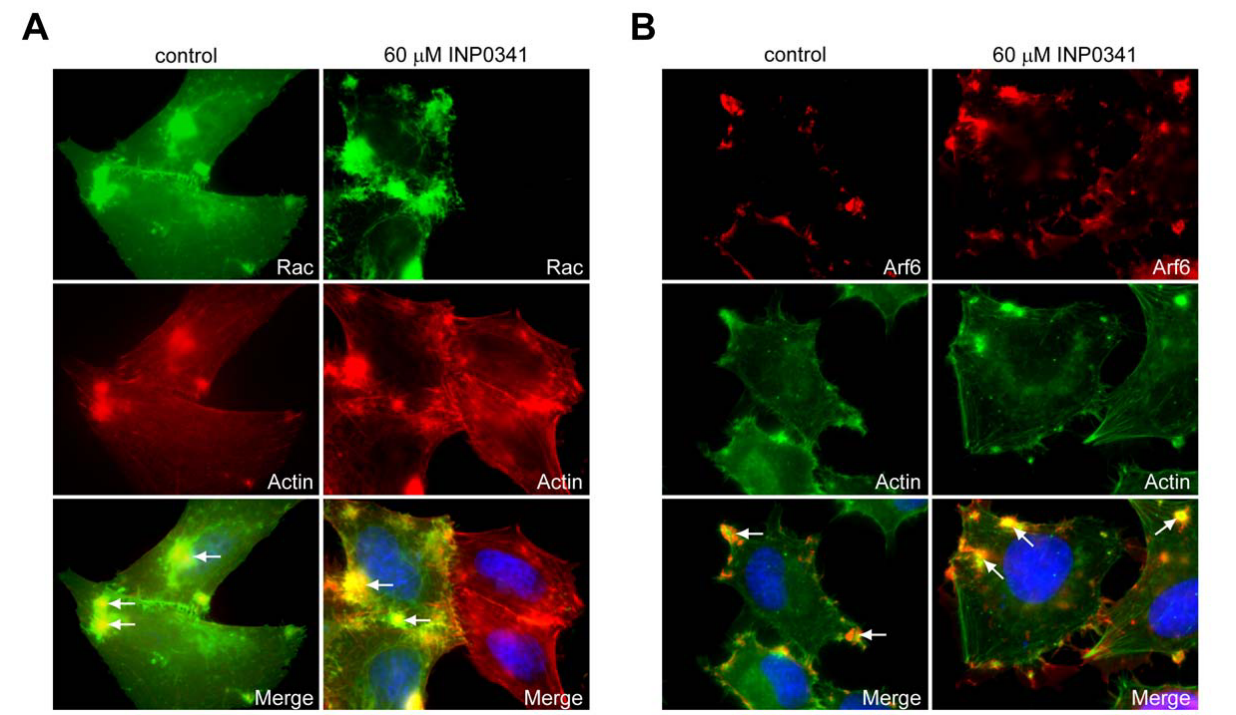
**Recruitment of Rac and Arf6 to *C. caviae *GPIC entry sites**. HeLa cells transfected with Rac-GFP (A) or Arf6-HA (B) for 24 h were infected with *C. caviae *GPIC in the presence of DMSO (control) or 60 μM INP0341. At 10 minutes p.i. cells were fixed and processed for immunofluorescence. Top panels show labelling for the small GTPases, middle panels show actin labelling and bottom panels show superimposition of the two images, as well as host cell nuclei stained with Hoechst 33342 (blue). Rac and Arf6 are recruited to sites of actin polymerization (arrows), both in control cells and in cells treated with INP0341.

The small GTPases Rac, Cdc42 and Arf6 are recruited to the sites of *C. caviae *GPIC entry, and their activity is needed for bacterial invasion [11,12]. HeLa cells were transfected with either Rac-GFP, Cdc42-GFP or HA-tagged Arf6 for 24 h before being infected with *C. caviae *GPIC. At 10 minutes p.i. cells were fixed and labelled for actin. Rac and Cdc42 were localized by the GFP signal; Arf6 was labelled with anti-HA antibodies. Rac-GFP (Fig. 3A), Arf6 (Fig. 3B) and Cdc42-GFP (data not shown) were found to be localized to the actin aggregates to the same extent in cells infected in the presence of INPs as in control cells. Therefore, INPs do not interfere with the recruitment of small GTPases to *C. caviae *GPIC entry sites, which strongly support the other observations that *Chlamydia *entry proceeds normally in drug treated cells.

## Discussion

Our data show that INPs do not inhibit the entry of *Chlamydia *into host cells. The efficiency of bacterial invasion has been investigated with two *Chlamydia *species, *C. trachomatis *L2 and *C. caviae *GPIC, and it was not modified in the presence of the drug. The normal recruitment of Rac, Cdc42 and Arf6 to *C. caviae *GPIC entry sites in the presence of INPs further indicates that INPs do not interfere with the mechanism of *Chlamydia *invasion.

Previously, we had reported a partial effect on *Chlamydia trachomatis *L2 entry in the presence of INP0400 [17]. This was based on the observation that treatment of the cells with 40 μM INP0400, for the first 3 hours of infection, resulted in a 40% reduction in the percentage of infected cells, compared to non-treated cells. We interpreted these data as a partial effect of the drug on bacterial entry. However, since we demonstrate here that *Chlamydia *invasion is not impaired by treatment with INPs, a more likely explanation is that other early events, following *Chlamydia *entry, are required for the onset of infection and are susceptible to the drugs. Indeed, *Chlamydia *genes expressed early in infection are needed to create a permissive environment for successful bacterial replication [21]. In particular, some of the Inc proteins, which are T3S substrates, are transcribed very early during infection and can be detected in the inclusion as early as 2–4 h p.i. [7].

In support of our results, Wolf et al. and Slepenkin et al. had reported that they were unable to inhibit *C. trachomatis *L2 entry in presence of INPs [18,19]. In the study of Wolf et al. the effect of drug on the EB translocated protein TARP, which probably plays a central role in the internalization process of *C. trachomatis *was examined. Upon host cell attachment, TARP is secreted in a type III dependent manner by *Chlamydia trachomatis *and becomes rapidly phosphorylated. Wolf et al., were unable to inhibit this early tyrosine phosphorylation of TARP in cells treated with another compound of the same family of INPs [18]. The lack of effect of INPs, which have been identified and described as type III secretion inhibitors, on *Chlamydia *entry is therefore surprising. Recent reports on the mode of action of INPs which we would like to discuss here, raise the question whether these drugs interfere with the actual translocation process of T3S substrates or rather inhibit at the level of transcription of T3S associated genes or assembly of the T3S machinery.

Earlier studies suggested that INPs might affect the translocation of type III substrates per se, and indeed, in *Yersinia*, careful analyses suggest that it is the case [22]. So far, the efficiency of INPs at blocking T3S in *Chlamydia *has been shown only for substrates secreted by RBs, and their target might be missing in EBs. In favour of this hypothesis is the observation that *Chlamydiae *genomes encode two homologues for the *Yersinia *lcrH chaperone for T3S system structural components, lcrH-1 and lcrH-2 [23]. These genes are in clusters that are differentially expressed during the developmental cycle. It was recently shown that transcription of lcrH-1, which is expressed late in the cycle, when EBs are forming, was inhibited by INP0341, while transcription of lcrH-2, which is expressed earlier in the cycle, was not [19]. Functional differences in the T3S apparatuses of EBs and RBs might therefore explain a difference in sensitivity to the type III secretion inhibitors. This would be consistent with our results and could explain the lack of effect of INPs on *Chlamydia *entry.

As an alternative, it is possible that INPs have a different mode of action on *Chlamydia *development than they have on *Yersinia*, and do not block the translocation of effectors per se. Importantly, the effect of INPs on chlamydial development is fully reversed by the addition of iron [19], while their inhibitory effect on *Yersinia *T3S is not (personal communication from Innate Pharmaceuticals AB). In this case, INPs might affect one of two requirements for effector protein secretion: (a) the assembly of functional secretion apparatuses or (b) the synthesis of the substrates recognized by the secretion machinery.

By acting on the formation of type III secretion apparatuses, INPs would only be effective when introduced while the apparatuses are being made, i.e. in the intracellular multiplication phase of *Chlamydia *development. In support of this hypothesis, recent data strongly suggest that, in the case of *Shigella*, INPs block assembly of the type III secreton [24]. In *Shigella*, INPs were only effective at inhibiting host cell invasion when added during growth, rather than during the infection step.

If, on the other hand, INPs inhibited the synthesis of type III secretion substrates, they would not affect entry either, because the effectors needed for this step are not newly synthesized during entry. INP0400 has been shown to inhibit the secretion of IncA and IncG proteins, which are produced during RB proliferation, and are rapidly translocated upon synthesis, as they are only weakly detected in RBs [25,26]. In contrast, Tarp and other potential T3S effectors participating in the entry event are at least partially stored in the RBs to be released by the EB form upon infection. Recent data show that the expression of some of the T3S genes (including genes coding for the secretion apparatus) is down-regulated by INP0341 [19]. Similarly, the *Yersinia *T3S system is down-regulated upon bacterial exposure to INPs [22] and it has also been shown in *Salmonella *that these compounds cause transcriptional silencing of the *Salmonella *pathogenicity island 1 [27]. It should be noted that if INPs act at a transcriptional level in *Chlamydia*, they might not affect the secretion of all effectors to the same extent. Therefore, at this stage INPs should only be used cautiously to assess the mechanism of secretion of a given chlamydial protein.

Down-regulation of transcription could perhaps also be due to feedback inhibition resulting from blocking T3S activity [24]. If, in *Chlamydia*, either the transcription of T3S associated genes or the assembly of the T3S machinery are inhibited, addition of the drugs at the end of one cycle of infection is expected to affect the next round of infection. This is exactly what was observed when looking at the progeny of *C. trachomatis *infected cells treated with INP0341 24 hours post infection [19]. In this experiment, although the inclusions formed upon late INP0341 treatment were as abundant as in control cells, there was a decrease in the infectious progeny, suggesting that EBs formed in the presence of INPs might be defective in their ability to secrete type III effectors. However, due to the asynchronicity of the *Chlamydia *developmental cycle, we can not definitively rule out that the decrease in the formation of infectious EBs when the drug is added late in the cycle is not due to the now well documented reduction of RB multiplication upon INP treatment.

## Conclusion

In the present study we demonstrate that small molecule inhibitors of *Yersinia *T3S have a strong inhibitory effect on *Chlamydia *growth but fail to inhibit *Chlamydia *invasion. INPs had no significant effect on *C. trachomatis *L2 and *C. caviae *GPIC entry into epithelial cells. Moreover, recruitment of actin and small GTPases to bacterial entry sites was not altered. These results suggest that in the presence of INPs pivotal events in early *Chlamydia *biogenesis following entry must be affected which could account for the observed inhibition of *Chlamydia *growth. The inability of INPs to interfere with the entry mechanism suggest that the drug might not affect the translocation process per se. We believe that the identification of the mode of action of INPs on type III secretion in genetically tractable bacteria will clarify this issue.

## Methods

### Cells, bacterial strains, antibodies and plasmids

HeLa cells were grown as described [11]. The *Chlamydia trachomatis *L2 strain 434 (VR-902B) was from the ATCC and the GPIC strain of *C. caviae *was obtained from Dr. R. Rank (University of Arkansas). Plasmids coding for HA-tagged Arf6, GFP-tagged Rac and GFP-tagged Cdc42 were kindly given by Drs. Ph. Chavrier (Institut Curie, Paris), G. Tran van Nhieu (Institut Pasteur, Paris) and E. Caron (Imperial College, London), respectively. The mouse anti-*Chlamydia *antibody (unlabelled and FITC-conjugated) was purchased from Argene, Biosoft. Alexa546-phalloidin, Alexa488-phalloidin, goat Alexa488-coupled anti-mouse antibody and Hoechst 33342 were from Molecular Probes. The mouse anti-EfTu antibody was a kind gift from Dr. YX Zhang, Boston, USA. Rat anti-HA antibody was from Roche and the TRITC-conjugated anti-rat antibody was from Jackson Immuno Research. Cy™-5-conjugated goat anti-mouse antibody was purchased from Amersham.

### INPs

Two salicylidene acylhydrazides, namely INP0400 and INP0341, were provided by Innate Pharmaceuticals AB, Umeå, Sweden. The compounds were dissolved in dimethyl sulfoxide (DMSO, Sigma) as 10 mM stock solutions and used at the concentrations indicated.

### *Chlamydia *entry assay

HeLa cells were infected with *C. trachomatis *L2 or *C. caviae *GPIC in the presence or absence of 60 μM INP0400 or INP0341 and centrifuged for 5 minutes at 770 *g *at room temperature. Cells were fixed 2.5 h later and extracellular and intracellular bacteria were labelled as described [11]. In brief, extracellular bacteria were labelled with anti-*Chlamydia *antibody followed by anti-mouse Cy™-5 antibody. The cells were then permeabilized in PBS containing 0.05% saponin and 1 mg/ml BSA and intracellular bacteria were labelled with FITC-conjugated anti-*Chlamydia *antibody. The number of extracellular and intracellular bacteria was counted in 15 fields, with an average of 75 bacteria per field, in two independent experiments. The efficiency of entry is expressed as the ratio of intracellular to total cell-associated bacteria (intracellular and extracellular).

### Immunofluorescence microcopy

To visualize the effect of the drugs on *Chlamydia *development, HeLa cells infected with *C. trachomatis *L2 or *C. caviae *GPIC were grown in presence of INPs (or DMSO for control) for 24 h, fixed, and labelled with anti-EfTu antibody followed by Alexa488-coupled goat anti-mouse antibody. DNA was stained with 0.5 μg/ml Hoechst 33342 in the mounting medium.

Recruitment of actin to bacterial entry sites was visualized with Alexa546-phalloidin in HeLa cells infected with FITC-labelled *C. caviae *in the presence or absence of 60 μM INP0341 as described [11]. To visualize Arf6 and Rac distribution, cells were transfected with HA-tagged Arf6 or GFP-tagged Rac. Hela cells were infected with *C. caviae *GPIC 24 h after transfection and spun for 5 minutes at 770 *g *at room temperature. At 10 minutes p.i. cells were fixed and labelled with Alexa546-phalloidin (GFP-Rac transfected cells) or Alexa488-phalloidin (Arf6-HA transfected cells). Arf6 was labelled with a rat anti-HA antibody (Roche, clone 3F10) followed by a TRITC-conjugated anti-rat antibody (Jackson Immuno Research). Immunofluorescence microcopy was performed with an epifluorescence microscope (Axiophot, Zeiss, Germany) attached to a cooled CDD camera (Photometrics, Tucson, AZ), using a 63× Apochromat lens.

## Competing interests

The authors declare that they have no competing interests.

## Authors' contributions

SM carried out all experiments. SM and AS designed the study and analyzed the data. SM, SN, BHN and AS wrote the manuscript. All authors read and approved the final manuscript.
